# Transcriptomic and Metabolic Profiling Reveal the Mechanism of Ovule Development in *Castanea mollissima*

**DOI:** 10.3390/ijms25041974

**Published:** 2024-02-06

**Authors:** Yanhong Cui, Xingzhou Ji, Yu Zhang, Yang Liu, Qian Bai, Shuchai Su

**Affiliations:** 1 College of Forestry, Beijing Forestry University, Beijing 100083, China; cuiyh@bjfu.edu.cn (Y.C.); jxz2000@bjfu.edu.cn (X.J.); buazhangyu@163.com (Y.Z.); 2 State Key Laboratory of Efficient Production of Forest Resources, Beijing 100083, China; 3 Beijing Advanced Innovation Center for Tree Breeding by Molecular Design, College of Plant Science and Technology, Beijing University of Agriculture, Beijing 102206, China; bualiuyang@163.com

**Keywords:** chestnut, fertile ovules, abortion ovules, HPLC-MS/MS, transcriptome

## Abstract

Ovule abortion, which is the main cause of empty burs in the Chinese chestnut, affects the formation of embryos and further reduces yield; therefore, it is important to study the mechanism of ovule abortion. In this study, we analyzed the transcriptomic and metabolomic data of ovules at critical developmental stages to explore the key regulatory networks affecting ovule development. The metabolites were enriched mainly in pathways involved in phytohormone signaling, energy metabolism, and amino acid synthesis in the endoplasmic reticulum. Analysis of the differentially expressed genes (DEGs) revealed that the *HSP* genes were significantly down-regulated during fertilization, indicating that this process is extremely sensitive to temperature. The hormone and sucrose contents of ovules before and after fertilization and of fertile and abortive ovules at different developmental stages showed significant differences, and it is hypothesized that that abnormal temperature may disrupt hormone synthesis, affecting the synthesis and catabolism of sucrose and ultimately resulting in the abortive development of Chinese chestnut ovules. At the pollination and fertilization stage of chestnuts, spraying with ethylene, ACC, and AIB significantly increased the number of developing fruit in each prickly pod compared to CK (water) treatment. These results indicated that both ethylene and ACC increased the rate of ovule development. This study provides an important theoretical molecular basis for the subsequent regulation of ovule development and nut yield in the Chinese chestnut.

## 1. Introduction

The ovule is one of the final structures formed in flowers; it contains relatively little tissue but undergoes complex developmental transformations necessary for reproduction [1]. Embryonic development is a very important step in the process of plant growth and development, but not all sexual reproduction processes in plants occur smoothly and produce reproductively capable offspring [2]. On the one hand, ovule abortion leads to a reduction in the seating rate, resulting in yield reductions [3,4]. On the other hand, ovule abortion severely restricts hybridization and cross-breeding and limits the proliferation, reproduction, introduction, and conservation of endangered species. The mechanism of ovule failure has long been a research hotspot, and in-depth studies have been carried out on many crops; however, research on non-wood-product forest trees started only recently. Therefore, there is an urgent need to analyze the mechanism through which ovule abortion promotes yield in these species.

Numerous studies have shown that ovule development is closely related to environmental factors, such as temperature, water, light, and pests [5]. It was found that, during the pollination and fertilization stages, excessively high or low temperatures could increase the ovule abortion rate [3,5]. For example, for every 1 °C increase in the global average temperature, maize yields are reduced by 10% [6]. Additionally, a sudden decrease in temperature before or after flowering was shown to be a key factor causing ovule abortion in *Brassica napus* [7]. The occurrence of diseases and insect pests causes abnormal photosynthesis in plant leaves, which leads to a lack of energy supply during the process of seed development or directly affects the pollen supply during the pollination stage, resulting in ovule abortion [8]. Light insufficiency affects the development and maturation of florets. After pollination, light insufficiency can also lead to insufficient photosynthesis, a severe lack of nutrients, and ovule abortion. Zhao et al. reported that shading treatment during the corn grain formation stage resulted in complete grain abortion [9]. Water stress not only affects plant growth but also affects embryo development. Under drought conditions, photosynthesis in maize is inhibited, resulting in large-scale abortion of top grains [10]. Postflowering irrigation can increase spring corn yields [11]. Therefore, further exploration of the environmental regulatory mechanisms involved in the precise induction of ovule development can provide effective methods for obtaining high and stable yields.

In recent years, studies based on herbaceous model plants have revealed that ovule development involves a complex gene regulatory network [1]. To date, thousands of genes related to ovule abortion have been identified, and these genes are involved mainly in fertility and hormone production [12]. Li et al. reported that *VvHDZ28* regulates salicylic acid (SA) biosynthesis during rice seed abortion [13]. Overexpression of *VvHDZ28* resulted in a seedless tomato fruit and increased SA content [13]. Moreover, overexpression of the *CWIN* gene increased seed weight, while silencing its expression led to seed dysplasia [14]. The ethylene-responsive transcription factor ethylene-insensitive 3-like (*EIL1*) was found to be involved in ovule senescence, and overexpression of this gene in tomatoes resulted in premature ovule failure [14]. It was reported that the *VvGA3ox* and *VvGA2ox* gene families affect ovule abortion by regulating the content of active gibberellins [15]. Down-regulation of the expression of the genes encoding the ATPase PEPC and GAPDH in oil tea causes abnormalities in the cellular energy metabolism system, thereby affecting ovule development [2]. Research on the embryonic development of woody plants has remained incomplete due to the limitations posed by variable growth environments, complex genetic backgrounds, and long growth cycles.

*Castanea mollissima* is a species of *Castanea* in Fagaceae that originated in China. It is one of the most popular nut species in China and has a long history of consumption; the annual output for this species in China accounts for more than 80% of the global output. Although the annual yield of the Chinese chestnut ranks first worldwide, the unit yield is relatively low. Problems occurring at any time during the embryonic development of chestnuts lead to the formation of empty chestnut bracts, resulting in chestnut yield reduction [16]. Successful fertilization and normal embryonic development are the key steps in ensuring optimal chestnut yield. Abortion at any stage during these processes reduces the seed setting rate and severely affects yield [17]. However, in the Chinese chestnut, the regulatory hormone affecting ovule abortion and the regulatory mechanism for the hormone are unknown. Therefore, in this study, we determined the critical periods of fertilization and embryo development by cytological observation of the ovaries and ovules after pollination. Then, we analyzed the transcriptomic and metabolomic data of ovules at different developmental stages after pollination to determine the key candidate genes and metabolic pathways that affect fertilization and embryo development in chestnuts.

## 2. Results

### 2.1. Morphological Development of Chestnut Bur Ovaries and Ovules

**Figure 1:**
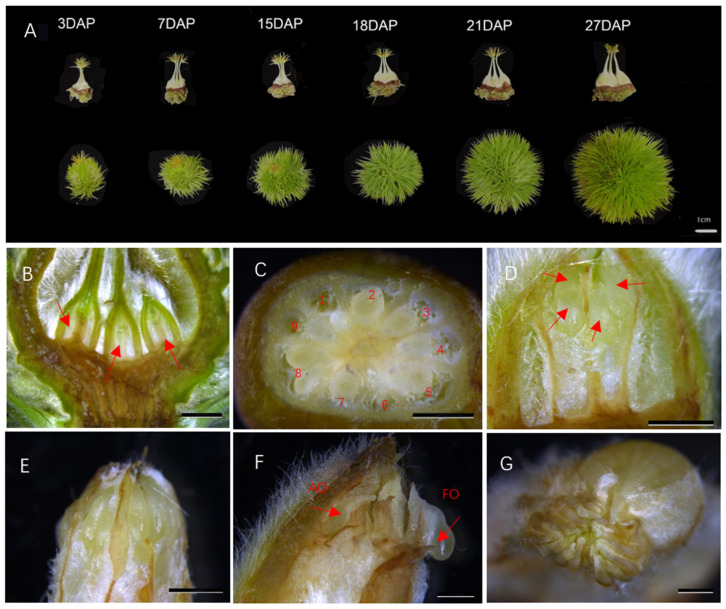
Morphological observations of chestnut development and abortive ovules. Note: (**A**) External morphology of the bract and ovary at different developmental stages. Scale bar is 1cm (**B**) Internal position of the ovary insertion in the bract; red arrows represent three ovaries. (**C**) Internal structure of the ovary; 1~9 represent nine embryo sacs. (**D**) Anatomy of the ovary prior to fertilization; red arrows represent pellicle ovules. (**E**) Developmental state of ovules at 18 DAP. (**F**) Developmental state of ovules at 21 DAP; AO represents abortive ovules, and FO represents fertile ovules. (**G**) Developmental state of ovules at 18 DAP. (**B**–**G**) are all on a scale bar of 2mm.

According to structural observations, each chestnut bur usually has three ovaries, each ovary has six to nine locules, and each locule has two inverted ovules, which are inserted on the placentation of the central axis through the diaphragm (Figure 1A–C). Preliminary research from our group revealed that the ovary expanded significantly, and the integument and nucellus began to develop at 7 days after pollination (DAP); the embryo sac matured at 15 DAP, but fertilization was not completed. At 18 DAP, the nucellus tissue in the pollinated ovule gradually disintegrated, and the embryo and milk nucleus began to divide (Figure 1E). The results showed that double fertilization was completed [18]. At twenty-one DAP, the morphology of fertile and abortive ovules in the same ovary began to differ. The abortive ovules were flat, while the fertile ovules were relatively full (Figure 1F). At twenty-seven DAP, the difference in size between fertile ovules and abortive ovules decreased, and browning began (Figure 1G). The external morphology of the chestnut spiny bract and ovary is shown in Figure 1A.

### 2.2. Transcriptome Analysis (Sequencing, Assembly, and Annotation) of Chestnut

Through transcriptome sequencing and analysis of 24 ovule samples at different developmental stages after pollination, a total of 165.18 Gb of clean data were obtained after quality control was completed. The clean data of each sample reached 5.98 Gb, and the percentage of Q30 bases was 91.54% or more. The clean reads of each sample were sequenced with the specified reference genome, and the alignment efficiency ranged from 92.05% to 96.11% (Table S1). Based on the comparison results, variable splicing prediction analysis, gene structure optimization analysis, and new gene discovery were carried out. A total of 5886 new genes were discovered, 4508 of which were annotated. PCA indicated that all the biological replicates were grouped together (Figure S1A), and correlation analysis revealed notable differences (Figure S1B). Based on the comparison results, gene expression was analyzed. The differentially expressed genes (DEGs) were identified according to the expression levels of genes in different samples. Functional enrichment analysis was performed, and the functions of the DEGs were annotated.

The unigene sequences were mapped to public databases using BLAST with an *E*-value cutoff of 10^−5^. Gene function was annotated based on the Nr, Nt, Pfam, KOG/COG, Swiss-Prot, KO, and GO databases. In total, 40,865 unigenes were matched to a sequence in at least one of the databases mentioned above (Table S2, Figure S1C).

### 2.3. DEGs in Ovules at Different Developmental Stages after Pollination

**Figure 2:**
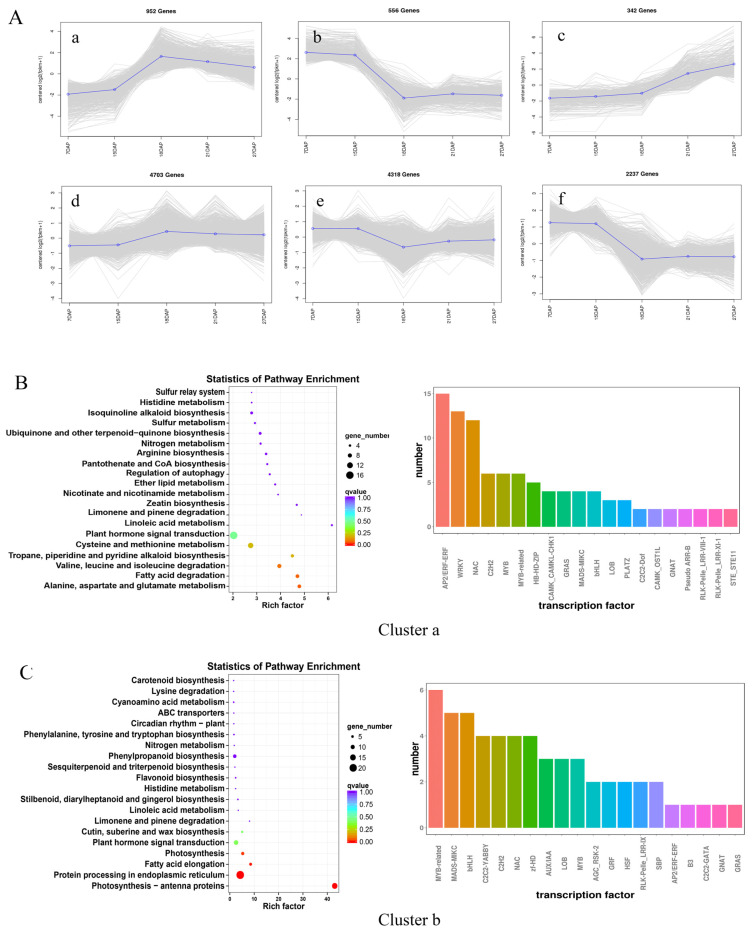
Analysis of gene expression patterns and KEGG enrichment and prediction of transcription factors during chestnut ovule development. Note: (**A**) K-means clustering analysis of gene expression patterns in chestnut ovule development; a–f represent different trends in expression. (**B**) KEGG enrichment analysis and transcription factor prediction of DEGs in cluster a. (**C**) KEGG enrichment analysis and transcription factor prediction of DEGs in cluster b. The color in the enriched bubble chart represents the *p* value, and the size of the point represents the number of enriched DEGs. The different colors in the bar graph represent the different transcription factors. The same applies below.

To analyze the DEG patterns, the expression data at different stages were centralized and standardized and then clustered by K-means. Based on the gene expression patterns, the DEGs in ovules at different developmental stages could be divided into six clusters (Figure 2A). Fifteen to 18 DAP is the critical period for fertilization. Clusters a, c, and d exhibited 952, 342, and 4703 up-regulated DEGs, respectively, and the DEGs in cluster a were more significantly up-regulated than those in clusters b and c. Clusters b, e, and f contained 556, 318, and 2237 down-regulated DEGs, respectively, and the DEGs in cluster b were more significantly up-regulated than those in clusters e and f.

Cluster a and cluster b contained the most significantly up-regulated and down-regulated genes at the critical stage of fertilization, respectively, indicating that they were positively associated with the fertilization process in chestnuts (Figure 2B). The 958 significantly up-regulated DEGs in cluster a contained 136 transcription factors, of which those of the *AP2/ERF-ERF* family were the most abundant, followed by *WRKY* and *NAC* transcription factors. The 556 DEGs in cluster b contained 75 transcription factors, of which *MYB*-related transcription factors were the most abundant, followed by *MADS-MIKC* and *bHLH* transcription factors (Figure 2C).

To better understand the characteristics of the DEGs and the differences, KEGG pathway enrichment analysis was conducted for the DEGs that were significantly up-regulated (cluster a) (Figure 2B) or significantly down-regulated (cluster b) in the critical period of fertilization. The top 20 enriched KEGG pathways are shown in Figure 2C. The 958 up-regulated DEGs were enriched mainly in the alanine, aspartate, and glutamate metabolism (ko00250); fatty acid degradation (ko00071); valine, leucine, and isoleucine degradation (ko00280); starch and sucrose metabolism (ko00010); and plant hormone signal transduction (ko04075) pathways. The 556 down-regulated DEGs were enriched mainly in the photosynthesis-antenna protein (ko00196), protein processing in the endoplasmic reticulum (ko04141), fatty acid elongation (ko00062), photosynthesis (ko00195), and plant hormone signal transduction (ko04075) pathways. It is hypothesized that the process of ovule fertilization is extremely sensitive to temperature and that excessively high or low temperatures may result in poor fertilization of chestnut ovules, leading to abortion. The DEGs in cluster a and cluster b were all enriched in the plant signal transduction pathway, indicating that hormones play an important role in the process of ovule fertilization.

### 2.4. Analysis of DEGs between Fertile and Abortive Ovules at Different Stages

By analyzing the transcriptome data of fertile and abortive ovules at different stages of fertilization, 8648, 2363, and 5225 DEGs were identified, of which 3877, 1259, and 2646 DEGs were up-regulated and 4771, 1068, and 2579 DEGs were down-regulated at the three stages (Table S3). To analyze the expression patterns of these DEGs, all the DEGs were subjected to KEGG enrichment.

**Figure 3:**
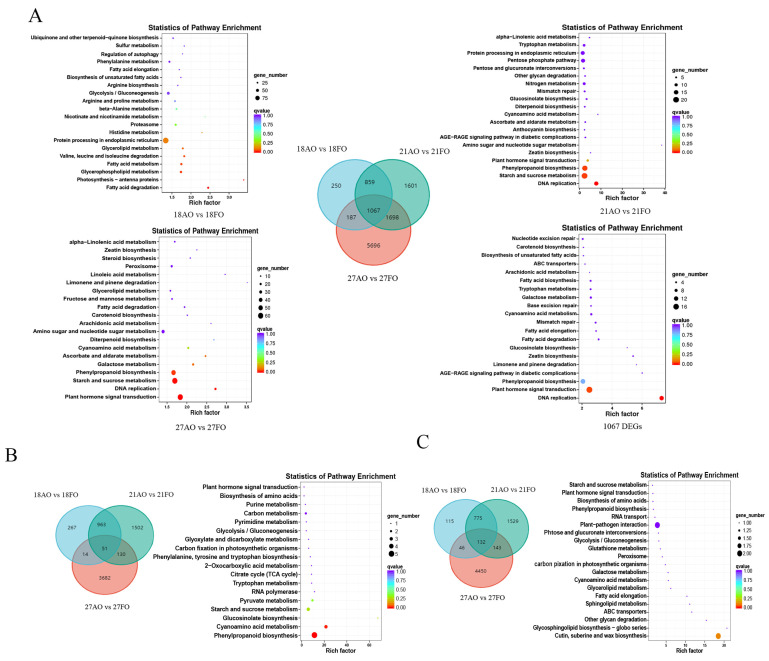
Identification and functional characterization of the DEGs between fertile and abortive ovules during the three stages Note: (**A**) KEGG enrichment analysis was performed for the DEGs between 18AO and 18FO, 21AO and 21FO, and 27AO and 27FO, for a total of 1067 DEGs. The Venn diagram depicts the shared and specific DEGs between the three compared groups. 18FO and 18AO represent 18-day postpollination development versus abortive ovules; 21FO and 21AO represent 21-day postpollination development versus abortive ovules; 27FO and 27AO represent 27-day postpollination development versus abortive ovules; and 1067 DEGs represent genes differentially expressed at three stages. (**B**) Venn diagram and KEGG enrichment analysis of DEGs co-up-regulated at all three developmental stages. (**C**) Venn diagram and KEGG enrichment analysis of DEGs co-down-regulated at three developmental stages.

At 18 DAP, the DEGs were significantly enriched (*p* < 0.05) in fatty acid degradation (ko00071); photosynthesis-antenna proteins (ko00196); glycerophospholipid metabolism (ko00564); fatty acid metabolism (ko01212); and valine, leucine, and isoleucine degradation (ko00280). At 21 and 27 DAP, the pathways associated with DEGs exhibiting significant enrichment between fertile ovules and abortive ovules were mostly the same, and the DEGs were enriched mainly in plant hormone signal transduction (ko04075), DNA replication (ko03030), starch and sucrose metabolism (ko00500), and phenylpropanoid biosynthesis (ko00940) (Figure 3A).

There were a total of 1067 DEGs in the fertile and abortive ovules at the three stages, of which 51 DEGs were co-up-regulated and 132 DEGs were co-down-regulated. The co-up-regulated DEGs were significantly enriched in phenylpropanoid biosynthesis (ko00940) and cyanoamino acid metabolism (ko00460) (Figure 3B). The co-down-regulated DEGs were enriched mainly in cutin, suberin, and wax biosynthesis (ko00073); glycosphingolipid biosynthesis-globo series (ko00603); and other glycan degradation (ko00511) (Figure 3C). A total of 1067 DEGs were enriched in the plant hormone signaling pathway, but the DEGs that were either co-up-regulated or co-down-regulated were not significantly enriched in this pathway, suggesting that hormones dynamically regulate the development of the ovule, that different hormones dominate at different stages of ovule development, and that multiple hormones synergistically regulate the development of ovules.

### 2.5. qRT-PCR and RT-PCR Validation of RNA-seq Data

**Figure 4:**
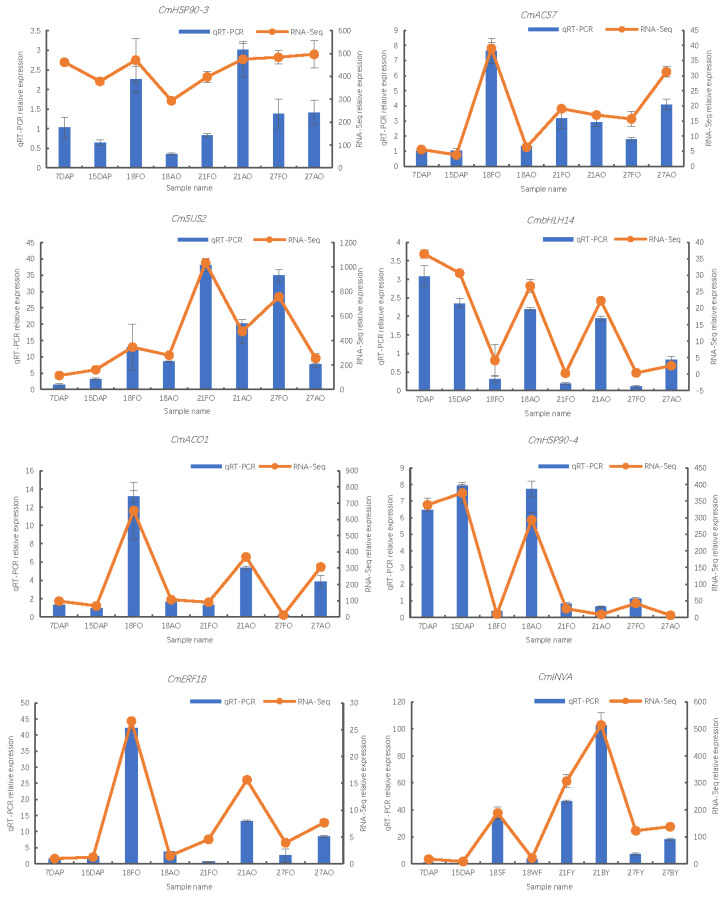
qRT-PCR analysis for 8 genes in all ovule samples. Note: Gene names correspond to the ID number: *CmACS7* (*CMHBY204224*), *CmERF1B* (*CMHBY224417*), *CmBHLH14* (*CMHBY225692*), *CmACO1* (*CMHBY214443*), *CmSUS2* (*CMHBY202474*), *CmHSP90-3* (*CMHBY204630*) *CmHSP90-4* (*CMHBY213842*), *CmINVA* (*CMHBY209168*). Vertical bars represent standard deviation (SD).

From the three pathways closely related to ovule development, namely plant hormone metabolism, glucose metabolism, and protein synthesis in the endoplasmic reticulum, we selected eight genes with significant expression differences, namely *CmACS7* (*CMHBY204224*), *CmERF1B* (*CMHBY224417*), *CmBHLH14* (*CMHBY225692*), *CmACO1* (*CMHBY214443*), *CmSUS2* (*CMHBY202474*), *CmHSP90-3* (*CMHBY204630*), and *CmHSP90-4* (*CMHBY213842*) for qRT-PCR and RT-PCR to verify the reliability of transcriptome data. The results showed that the qRT-PCR and RT-PCR results were consistent with the trend of expression levels detected by RNA-seq, which proved that the data were reliable (Figure 4 and Figure S2).

### 2.6. Metabolome Profiling

**Figure 5:**
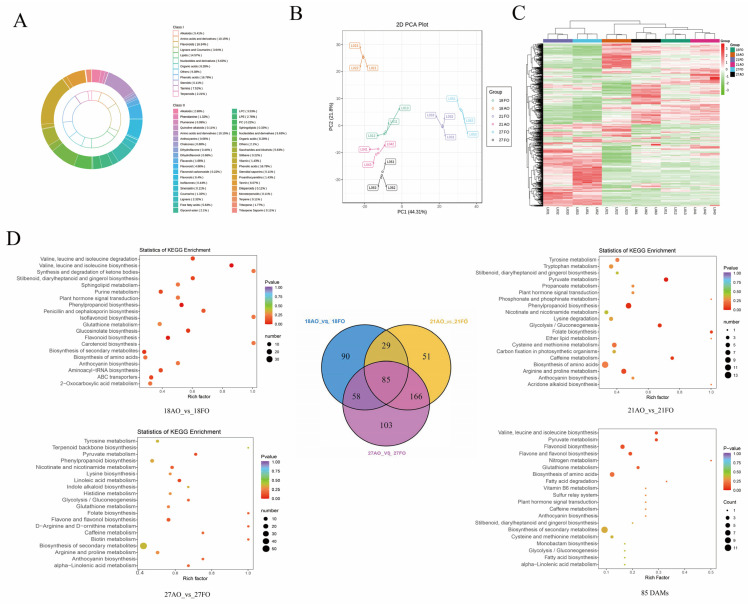
Metabolite profiles and functional characterization of the differentially accumulated metabolites (DAMs) of fertile and abortive ovules. Note: (**A**) Principal component analysis plot. (**B**) Heatmap of metabolite clustering. (**C**) Metabolite classification ring chart. (**D**) KEGG enrichment analysis of the DAMs between 18AO and 18FO, 21AO and 21FO, 27AO and 27FO, and 85 DAMs in all three developmental stages; Venn diagram depicting the shared and specific metabolites among the three compared groups of samples.

To understand the molecular mechanisms underlying the differences between fertile and abortive ovules, ovules at three different developmental stages were collected. A total of 906 metabolites were detected via the UPLC-MS/MS detection platform, and these metabolites were mainly classified into 11 categories. Among them, phenolic acids (16.78%) accounted for the largest proportion of metabolites, followed by amino acids and their derivatives (10.15%) (Figure 5A). To evaluate the sample similarity and identify correlated metabolic changes, PCA was performed with all 906 metabolites (Figure 5B). Principal components 1 and 2 (PC1 and PC2) explained 44.31% and 21.8% of the variation, respectively. Notably, the samples were well separated by the combination of PC1 and PC2. Additionally, a heatmap analysis of all the samples is shown in Figure 5C, where the difference between the fertile ovules and abortive ovules was significant at 21 and 27 DAP. Overall, these results suggested that the metabolic profiles of the fertilized ovules and abortive ovules were markedly different.

### 2.7. Identification of the Differentially Accumulated Metabolites (DAMs) between Fertile and Abortive Ovules

The DAMs between pairs of sample groups (18 AO vs. 18FO, 21AO vs. 21FO, and 27AO vs. 27FO) were determined based on VIP ≥ 1 and FC ≥ 2 or FC ≤ 0.5. As expected, significantly high numbers of metabolites were differentially accumulated between the compared samples, including 266, 328, and 417 DAMs in 18 AO vs. 18FO, 21AO vs. 21FO, and 27AO vs. 27FO, respectively (Table S4). These results showed that the more advanced the development of the zygote is, the more complex the metabolic pathways involved become. The top enriched KEGG terms among the DAMs detected for all the compared samples were biosynthesis of secondary metabolites, phenylpropanoid biosynthesis, carbohydrate metabolism (energy metabolism), fatty acid biosynthesis and degradation, flavone and flavonol biosynthesis, amino acid biosynthesis and metabolism, flavonoid biosynthesis, and plant hormone signal transduction (ko04075) (Figure 5D).

Comparative analysis of the three groups of DAMs among the fertile and abortive ovule samples revealed 85 common metabolites, including 19 co-down-regulated and 35 co-up-regulated compounds. These DAMs may include potential metabolites associated with ovule development. KEGG enrichment analysis was carried out on these common DAMs, and the results revealed enrichment in mainly amino acid biosynthesis and metabolism, fatty acid biosynthesis and degradation, flavonoid biosynthesis, glycolysis/gluconeogenesis, and plant hormone signal transduction (ko04075) (Figure 5D).

**Figure 6:**
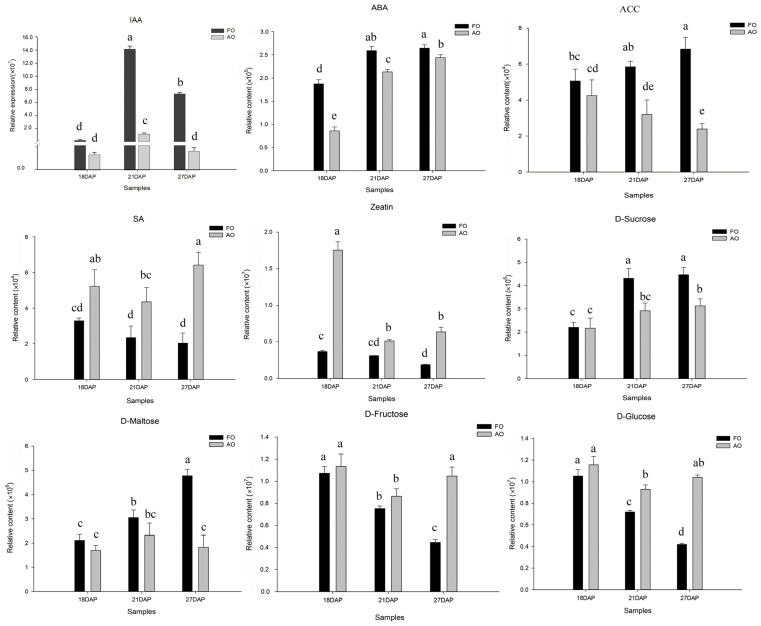
Hormone and sugar contents of fertile and abortive ovules at different developmental stages. Note: Different lowercase letters indicate a significant difference at the 0.05 level, and the same letter indicates no significant difference. Vertical bars represent the standard deviation (SD).

In summary, ovules undergo large changes in hormone and sugar levels during development. To understand the differences between sugars and hormones in developing and abortive ovules, we analyzed sugars and hormones separately. Among the sugars, sucrose and maltose exhibited higher levels in developing ovules than in abortive ovules, whereas glucose and fructose exhibited higher levels in abortive ovules than in fertile ovules. Among the hormones, indole-3-acetic acid (IAA), abscisic acid (ABA), and the precursor of ethylene synthesis, 1-aminocyclopropane-1-carboxylic acid (ACC), exhibited higher levels in developing ovules than in abortive ovules, whereas SA and zeatin (ZT) exhibited lower levels in developing ovules (Figure 6). Ovule development is dynamically regulated by a variety of hormones, and the secondary process requires energy from the decomposition of sugar species (Figure 6).

### 2.8. Integrative Analysis of the Transcriptome and Metabolome

**Figure 7:**
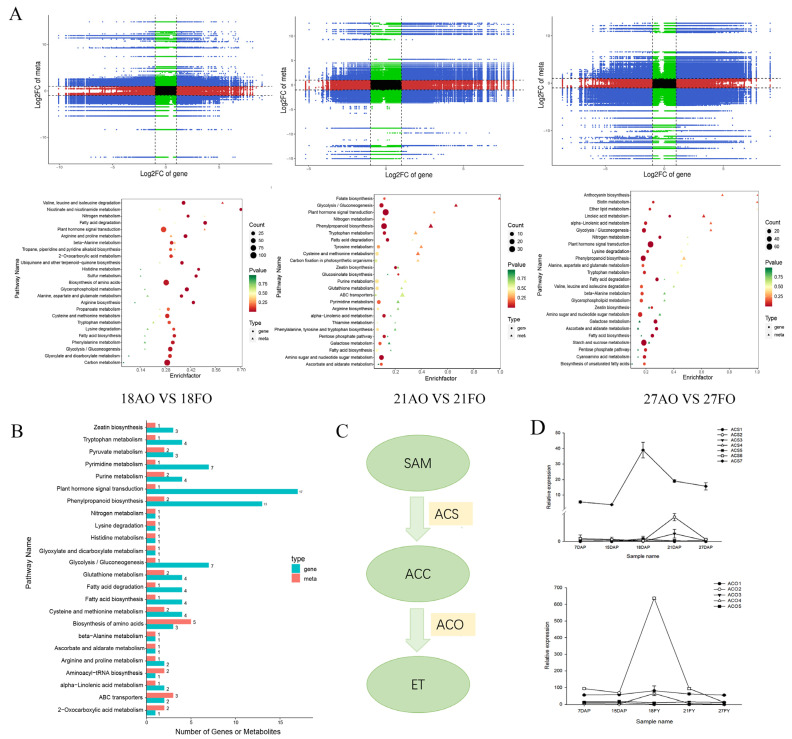
Correlation analysis of genes and metabolites detected in different developmental stages. Note: (**A**) Nine-quadrant plot for correlation analysis and KEGG enrichment analysis of the three stages. (**B**) KEGG classification showing enriched pathways. The horizontal coordinates represent the number of DAMs and DEGs enriched in the pathway, the vertical coordinates represent the KEGG pathway name, and the red and green bars represent the metabolome and transcriptome, respectively. (**C**) Pathway of ethylene biosynthesis. (**D**) Relative expression of *CmACS* and *CmACO* during ovule development.

Correlation analysis of genes and metabolites detected in each differential grouping. The nine-quadrant results showed that 8384 (18 DAP), 3725 (21 DAP), and 5276 (27 DAP) genes had PCC values ≥ 0.8, and 274, 349, and 450 metabolites were identified in the third and seventh quadrants at 18, 21, and 27 DAP, respectively. A heatmap analysis of DEGs and DAMs with a PCC > 0.8 was also prepared to clearly visualize their clustering characteristics (Figure 7A).

To identify key pathways that potentially contribute to ovule abortion in the ovaries of chestnuts, we conducted KEGG pathway enrichment analysis of the DEGs and DAMs identified between fertile and abortive ovules (Figure 7A). The results showed that the DEGs and DAMs of the three stages were all enriched in a variety of amino acid metabolism, plant hormone signal transduction, and energy metabolism pathways (including fatty acid degradation, glycolysis and gluconeogenesis, and starch and sucrose metabolism).

The results of KEGG statistical analysis of DEGs and DAMs in the three stages showed that the greatest number of DEGs enriched in plant signal transduction pathways was 17, and the only DAM was IAA. The second most enriched pathway was that of phenylpropanoid biosynthesis, with 13 DEGs and two DAMs (trans-5-O-(p-coumaroyl) shikimate, trans-5-O-coumaroyl ferulate; scopoletin-7-O-glucoside (scopolin), scopoletin-7-O-glucoside) enriched in this pathway. The pathway associated with the most DAMs was the amino acid synthesis pathway, with five metabolites (L-glutamine, L-histidine, 2-isopropylmalic acid, 3-isopropylmalic acid*, and S-adenosyl-L-methionine (SAM, ACC precursor)) and three genes (*CMHBY201597* (*PFK3*), *CMHBY206729* (*PGK*), and *CMHBY214996* (*PAT1*)) enriched in this pathway. In addition, seven DEGs were enriched in the pathways of sugar catabolism and sugar isomerization, as well as one metabolite (glucose) (Figure 7B).

Recent studies have shown that SAM not only acts as a methyl donor for cells but also participates in the biosynthesis of polyamines, ethylene, and malic acid [19]. SAM is converted to ACC via a process catalyzed by ACC synthase (ACS), and ACC is subsequently oxidized to ethylene by ACC oxidase (ACO) (Figure 7C). Analysis of the ACC content in the developing ovules revealed that the ACC content in the three stages was greater in the fertile ovules than in the abortive ovules (Figure 6). Therefore, it was inferred that ACC or ethylene plays an important role in the development of ovules.

Analysis of the expression of the *ACS* and *ACO* genes revealed that the expression trends of *CmACS7* (*CMHBY204224*) (Figure 7D) and *CmACO1* (*CMHBY214443*) (Figure 7D) were largely the same during ovule development, and their expression was significantly up-regulated from 15 to 18 DAP, the critical period for fertilization. The rapid down-regulation of gene expression after the completion of fertilization indicated that fertilization activated the expression of *ACS* and *ACO*, and the simultaneous up-regulation of the two genes also indirectly indicated that the ethylene content was significantly increased during this time. The down-regulated expression of these genes after the completion of fertilization may be attributed to the functions of *CmACS7* and *CmACO1* only at this stage.

### 2.9. Effect of Growth Regulators on the Development of Chestnut Ovaries

Differential analysis of fruiting among the different chestnut treatment groups revealed that all the treatments yielded 0% empty burs at maturity because all the seeds in the burs, after being aborted, fell out before maturity. With increasing ETH concentration, the single-fruit weight, the number of nuts in each bur, and the bur set rate tended to increase and then decrease, and all the indexes were significantly lower than those of the control group when the concentration reached 400 mg/L; however, the differences in the external morphology were not significant. The effects of spraying AIB on the single-fruit weight of chestnut plants and on the number of nuts on each bur were not significant, but concentrations up to 400 mg/L significantly reduced the bur set rate. Spraying 50 mg/L ACC significantly increased the number of developing ovaries of chestnuts, but when the concentration exceeded 200 mg/L, it extremely significantly reduced the fluffing rate of chestnuts.

## 3. Discussion

In this study, by sequencing the transcriptomes of chestnut ovules at different developmental stages after pollination, a total of 123.15 Gb of high-quality clean reads were obtained, with Q30 > 91.54%, which met the requirements for subsequent analysis. To analyze the DEG patterns in chestnuts during pollination and fertilization, the transcriptome data of ovules at different developmental stages were pooled, normalized, and subsequently clustered via K-means. Based on the gene expression patterns, the DEGs in ovules at different developmental stages could be categorized into six clusters. A total of 952 DEGs that were significantly up-regulated (cluster a) and 556 DEGs that were significantly down-regulated (cluster b) were analyzed. Subsequently, transcriptome and metabolome sequencing and analysis of fertile and abortive ovules from three developmental stages were performed, and the pathway enrichment results for the DEGs and DAMs of fertile and abortive ovules from the three developmental stages were basically consistent with the transcriptome data obtained during pollination and fertilization. These findings indicate that the DEGs enriched in these metabolic pathways play important roles in the development of chestnut ovules and that further research should be carried out on these pathways in the future.

### 3.1. Carbohydrate Metabolism Analysis

**Figure 8:**
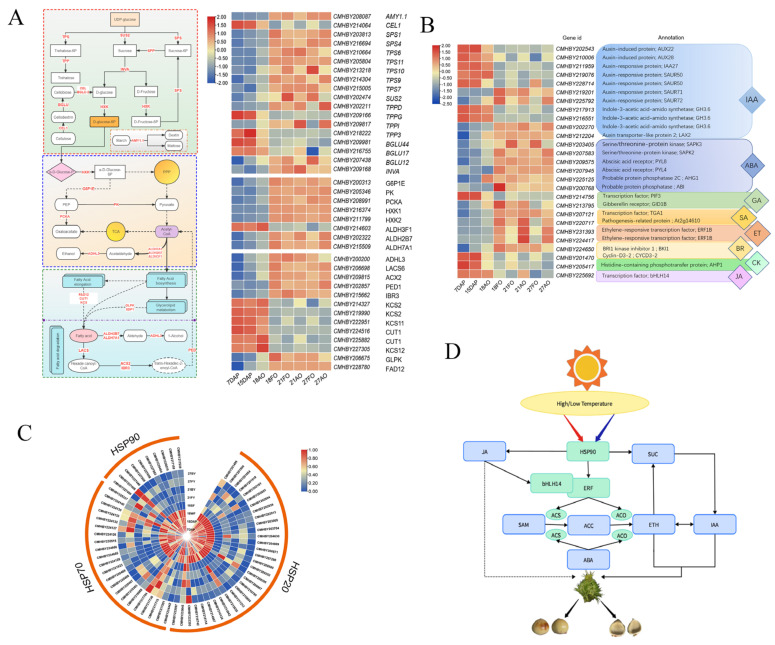
Key gene regulatory network and expression heatmap. (**A**) Carbohydrate metabolic pathway map and heatmap of key gene expression. (**B**) Heatmap of hormone-related gene expression. (**C**) Heatmap of heat stress protein-encoding gene and transcription factor expression. (**D**) Regulatory network of genes and their metabolites under the influence of temperature.

Carbohydrates can be used as energy substances during ovule development but also have osmoregulatory and catalytic activities and other functions. The soluble sugar and starch contents of aborted maize kernels were lower than those of normal kernels, and it was hypothesized that this difference might be due to the insufficient supply of organic nutrients, which leads to severe seed abortion at the top of the maize cob [20]. In a study of ovule septation in *Oryza sativa*, the soluble sugar content in normal ovules increased continuously from 12 to 14 days after anthesis, while that in septic ovules increased and then decreased. It was hypothesized that the early stage of ovule development in *O. sativa* (when fertilization was completed and the endosperm was in the period of free nucleus) might be related to the soluble sugar content. In addition to ovule development being related to the accumulation of organic matter, weak synthesis of organic matter can also cause ovule failure [21]. In our study, the metabolomic data analysis revealed differences in the contents of different sugars, with sucrose and maltose contents being greater in fertile ovules than in abortive ovules, while fructose and glucose contents were lower in fertile ovules than in abortive ovules. Analysis of the transcriptome data revealed that fertilization not only activated genes related to carbohydrate catabolism (*AMY*, *SPS*, *SUS, INVA*, *PK*) but also promoted the up-regulation of the expression of genes related to carbohydrate synthesis (*TPS*, *TPPD*, *HXK*) (Figure 8A). The DEGs were enriched in carbohydrate-related pathways such as starch and sucrose metabolism, glycolysis and gluconeogenesis, and fatty acid anabolism, both during ovule development and in fertile and abortive ovules (Figure 7A). This difference may be related to the fact that fertilized ovule development requires a large amount of energy, and these metabolic pathways provide a large amount of low-molecular-weight soluble sugars for ovule development. Alterations in any of these pathways can cause abnormalities in ovule development, leading to abortion.

### 3.2. Plant Hormone Signal Transduction Analysis

Plant development cannot be achieved without the synergistic regulation of several hormones, which play important roles in synchronizing fertilization and fruit growth. Early septation of fertilized ovules prevents the development of pea fruit, but the application of several plant growth regulators restores fruit growth, indicating that hormones are essential for fruit development [22]. Early septation of fertilized ovules prevents the development of pea fruit, but the application of several plant growth regulators restores fruit growth, indicating that hormones are essential for fruit development [20]. Ovule differentiation and growth are dependent on pollination and fertilization, during which development is inhibited prior to pollination and fertilization, and after pollination and fertilization, multiple signals are activated within the ovule to promote early ovule development [23]. In our study, transcriptome data analysis revealed significant differences in the expression of genes related to phytohormone signaling before and after fertilization. Metabolomic data also revealed a more pronounced difference in chestnut ovules after fertilization than in unfertilized samples (18AO vs. 18FO), where the levels of IAA, ABA, and ACC were greater in fertilized samples than in unfertilized samples, while the opposite was true for SA and zeatin. These findings suggested that fertilization activated IAA, ABA, and ACC signaling, thereby promoting early ovule development. This finding was in general agreement with the findings of Figueiredo and Köhler [23].

Ethylene, an important hormone involved in the entire plant life cycle, plays several important roles in the early development of the floral organ [24]. Pollination and fertilization accelerate ethylene biosynthesis, which in turn is involved in subsequent developmental processes, including petal senescence and ovary growth [25]. It has also been demonstrated that ethylene alone is not sufficient to trigger ovary development or ovule differentiation; a growth hormone is needed for ovary/ovule development [26]. Exogenous growth hormone treatment induces ethylene production and initiates most pollination-regulated developmental processes, and spatiotemporal regulation of ethylene and growth hormone levels in flowers may be important for ovary/ovule development after fertilization [27]. Based on the transcriptomic and metabolomic data from this study and the findings of previous studies, it is hypothesized that the development of chestnut ovules is also synergistically regulated by multiple hormones. The levels of IAA and ACC were greater in postfertilization ovules than in prefertilization ovules and were greater in developing ovules than in abortive ovules (Figure 5). The changes in the expression of growth hormone and ethylene-related genes, such as *IAA*, *ACS*, and *ERF1B*, were consistent with the changes in metabolites, suggesting that fertilization activated the expression of IAA- and ACC-related genes (Figure 8B), which contributed to the increase in their contents. This finding is different from the previous conclusion that pollination induces an increase in the contents of these genes, which may be related to the slow growth of pollen tubes after pollination of chestnut ovules, which requires a long period of 18 days to reach the bead pore and complete fertilization. In the ethylene biosynthesis and signaling pathway, ACS and ACO are important rate-limiting enzymes; i.e., the activities of ACS and ACO indirectly respond to the rate of ethylene synthesis [24]. The expression of the key genes *CmACS7* and *CmACO1*, which are involved in the regulation of these two enzymes, increased rapidly during the fertilization period (15 DAP-18 DAP) and decreased rapidly after the completion of fertilization (Figure 7C), suggesting that ethylene plays an important regulatory role only during fertilization, which is key for normal ovule development. In our study, upon spraying different concentrations of ethylene, ACC, and AIB, we found that low concentrations of ethylene and ACC significantly increased the number of developing nuts in the prickly bracts, suggesting that both low concentrations of ACC and ethylene promoted the development of the chestnut ovary, while high concentrations of both ACC and ethylene were detrimental to ovary development (Table 1).

### 3.3. Effect of Temperature on Ovule Development

Temperature plays an important role in the process of plant growth and development; when the ambient temperature exceeds the temperature range to which the plant is adapted, the growth and development of the crop are affected [28]. Studies have shown that the temperature requirements for plant flowering are extremely strict, and temperature stress can cause delayed or early flowering [29]. The exposure of corn to high temperatures in the reproductive growth stage affects the differentiation of female and male ears, reducing yields; high temperatures during flowering affect the development of grains, reducing the number of grains in the ear. It has also been found that the heat sensitivity of rice seeds is greatest in the first two days after fertilization and tends to decrease with the developmental process after ensiling [30]. Heat stress induces protein misfolding, and the accumulation of heat shock proteins (HSPs) plays an important role in cellular defense against temperature stress and other stresses [31]. In the present study, the vast majority of the heat stress protein-like genes, namely, *HSPs*, were found to be differentially expressed after fertilization in chestnuts, with significantly greater expression before fertilization than after fertilization (Figure 8C). These findings indicate that the fertilization of chestnut plants suppressed the expression of *HSP* genes and suggest that the process of fertilization, or the predevelopment period of the ovule after the completion of fertilization, is temperature-sensitive and that abnormal changes in temperature are detrimental to the development of chestnuts after fertilization and can lead to abortive ovule development. These results are consistent with the findings of a previous study in rice [30], where some phenotypic responses, such as changes in seed size and weight, persisted until maturity, although the applied stress was transient. The expression of most of the *HSP* genes in developing ovules was greater than that in abortive ovules, suggesting that developing ovules have a stronger ability to respond to stress than abortive ovules and that HSPs play an important role in ovule development.

Temperature anomalies reduce plant productivity and alter plant phenology and physiological responses. In response to temperature anomalies, plants have evolved multiple complex mechanisms, including hormone signaling pathways, to sense temperature stimuli and acquire stress tolerance. Ethylene is an essential gaseous hormone for plant growth and development and tolerance to various abiotic stresses, including temperature stress (high/low temperature) [32]. It has been demonstrated that ethylene production in response to temperature stress is species- and stage-specific [33]. Two ethylene peaks were detected in several plants during and after pollination and fertilization. A study on pea plants showed that heat induced ethylene production in the ovary prior to pollination but suppressed ethylene production in the ovary, stigma/stylus, and petals after fertilization [34]. Multiple ET biosynthesis and signaling genes, including *ACS3* and *ACS11*, *ERF1*, *ACO1*, and *ACO4*, are induced by heat stress [19]. Exogenous application of ACC improved freezing resistance in tomatoes, tobacco, and *Arabidopsis* [27]. In this study, temperature-related genes (*HSPs* and *HSFs*) were significantly differentially expressed after fertilization; most of them were significantly down-regulated after fertilization, and genes related to ethylene synthesis and response (*ACS7*, *ACO*, and *ERF1B*) were significantly up-regulated at this stage, which suggested that ACC and ET may be negatively regulated in response to changes in temperature and in response to changes in *HSP* genes after fertilization. This may be similar to the findings in pea plants [34].

Plants respond to external environmental stresses through the coordinated action of multiple hormones. Based on the results of this study combined with the results of previous studies, the regulatory network of chestnut ovule development was hypothesized to function as follows: fertilization inhibits the expression of thermostimulant protein genes, which makes chestnut ovules extremely sensitive to temperature changes, and temperature abnormalities (either high or low) affect the expression of ethylene synthesis genes (*ACS*, *ACO*) [33], which modulates the production of ethylene in ovules and subsequently influences their normal development. IAA and ABA promote ethylene synthesis; JA can inhibit the activity of *ERF*, a core transcription factor of the ethylene signaling pathway, by activating *MYC*, a *bHLH-type* transcription factor, which in turn affects ethylene production [35]; and SA reduces heat stress-induced high ET production and ACS activity [36]. JA promotes the synthesis of ethylene through sucrose [35]. In summary, the development of chestnut ovules is a complex process in which multiple metabolic pathways jointly regulate the development of chestnut ovules (Figure 8D). Our study demonstrated that spraying appropriate concentrations of ACC and ETH can significantly increase the number of developing ovaries in chestnuts, and further validation of other regulatory networks at the physiological and molecular levels will follow.

## 4. Materials and Methods

### 4.1. Plant Materials

The plant materials were obtained from the National Chestnut Breeding Base of Weijinhe, Zunhua City, Hebei Province (117°45′11″ E, 40°21′22″ N), and the mother plants were *C. mollissima* ‘Zunhuaduanci’, pollinated by *C. mollissima* ‘Zibo’, and *C. mollissima* ‘Dongling’ trees that were 15 years old. In the treatment groups, the plants were either bagged without pollination or subjected to full artificial pollination during the flowering stage. Ten trees were included in each treatment group, and 50 burs were selected from each tree. For the group subjected to bagging without pollination, the female flower clusters were covered with sulfuric acid paper bags on June 3 to isolate pollen, and the bags were removed when pollination of the male inflorescences in the chestnut garden was completed. To achieve adequate manual pollination, artificially assisted pollination was carried out on June 15 when the stigmas were tilted at 30°–45° (optimal pollination period), and freshly collected mixed pollen and inactivated pollen (exposed to 4 h of ultraviolet light) were gently applied onto the stigmatic clusters of bagged female flowers using a brush at 11:00 AM. This process was repeated at the same time the next day to ensure thorough pollination [37]. The day of pollination was designated 0 days after pollination (0DAP).

Fertilization of chestnut ovules was completed at 18 DAP (fertilization occurred between 15 and 18 DAP); however, at this time, fertile ovules and abortive ovules could not be distinguished from each other by appearance; thus, chestnut ovules that had been given inactivated pollen were collected as abortive chestnut samples at 18 DAP (18AO), and chestnut ovules that had been given fresh pollen were collected as developing ovules (18FO). After the fertile and abortive ovules could be distinguished from each other by appearance at 21 DAP, fertile ovules (21FO, 27FO) and abortive ovules (21AO, 27AO) were collected for transcriptional and metabolic assays, respectively. The stripped ovule samples were packed in 1.5 mL freezing tubes, and three replicates of each sample were examined for a total of 24 samples. These samples were quickly frozen in liquid nitrogen after plate stripping and then stored in an ultralow-temperature freezer at −80 °C for subsequent transcriptomic and metabolomic assays.

### 4.2. RNA Extraction, Library Construction, and Transcriptome Sequencing

Twenty-four libraries representing eight chestnut samples and three replicates were constructed for transcriptome sequencing. RNA extraction, quantification, and transcriptome sequencing were performed as described in detail previously [38]. The library preparations were sequenced on an Illumina platform, and paired-end reads were generated by Biomarker Technologies Co. (Beijing, China).

### 4.3. Transcriptome Data Analysis

DESeq2 was used to perform differential expression analysis of the two groups. DESeq2 provides statistical procedures based on a negative binomial distribution model to identify differential expression in digital gene expression data. Benjamini and Hochberg’s method for regulating the false discovery rate was applied to the derived *p* values. Genes with an adjusted *p* value < 0.01 according to DESeq2 were considered differentially expressed.

### 4.4. Quantitative Analysis of Metabolites

Ovules were selected at 18 (18FO, 18AO), 21 (21FO, 21AO), and 27 (27FO, 27AO) DAP for widely targeted metabolomic assays. Sample preparation, extract analysis, metabolite identification, and quantification were performed at Wuhan MetWare Biotechnology Co., Ltd. (Wuhan, China) (www.metware.cn) (accessed on 16 March 2021) following their standard procedures and as previously described in detail by Yuan et al. [39].

The sample extracts were analyzed using a UPLC-ESI-MS/MS system (UPLC, SHIMADZU NexeraX 2; MS, Applied Biosystems 4500 Q TRAP). Sample measurements were performed with a gradient program that employed the starting conditions of 95% A (pure water with 0.1% formic acid), 5% B (acetonitrile with 0.1% formic acid). Within 9 min, a linear gradient to 5% A, 95% B was programmed, and a composition of 5% A, 95% B was kept for 1 min. Subsequently, a composition of 95% A, 5.0% B was adjusted within 1.10 min and kept for 2.9 min. The flow velocity was set as 0.35 mL per minute; the column oven was set to 40 °C; the injection volume was 4 μL. The effluent was alternatively connected to an ESI-triple quadrupole-linear ion trap (QTRAP)-MS.

The procedure of sample extraction on the machine is as follows: firstly, grind ovule samples fully with liquid nitrogen. Dissolve 100 mg of lyophilized powder with 1.2 mL 70% methanol solution, vortex 30 s every 30 min for 6 times in total, place the sample in a refrigerator at 4 °C overnight. Following centrifugation at 12,000 rpm for 10 min, the extracts were filtrated (SCAA-104, 0.22μm pore size; ANPEL, Shanghai, China) before analysis.

Positive/negative ion mode of AB4500 Q TRAP mass spectrometer (AB SCIEX, Tokyo, Japan), multiple reaction monitoring mode (MRM mode) was used. The ESI source temperature 550 °C; ion spray voltage (IS) 5500 V (positive ion mode)/−4500 V (negative ion mode); ion source gas I (GSI), gas II (GSII), curtain gas (CUR) were set at 50, 60, and 25.0 psi, respectively; the collision-activated dissociation (CAD) was high. QQQ scans were acquired as MRM experiments with collision gas (nitrogen) set to medium. DP and CE for individual MRM transitions were done with further DP and CE optimization. A specific set of MRM transitions were monitored for each period according to the metabolites eluted within this period.

### 4.5. Metabolite Data Analysis

The statistics function prcomp in R-4.1.2 (www.r-project.org, accessed on 3 December 2023) was used for unsupervised principal component analysis (PCA). Prior to unsupervised PCA, the data were scaled using the unit variance. While Pearson correlation coefficients (PCCs) between samples were determined using R’s cor function and are displayed as heatmaps, the findings of the samples’ hierarchical cluster analysis (HCA) and their metabolites are displayed as heatmaps with dendrograms. The R package heatmap was used for both HCA and PCC. The color spectrum represents the adjusted signal intensities of metabolites (unit variance scaling) for HCA.

Metabolites that were significantly differentially expressed across the groups were indicated by both an absolute log2 (fold change, FC) greater than 1 and a variable importance in projection (VIP) greater than or equal to 1. The VIP values, taken from the OPLS-DA findings that included score plots and permutation plots, were produced using the R package MetaboAnalystR. Prior to conducting OPLS-DA, the data were log-transformed (log2) and mean-centered. To avoid overfitting, a permutation test with 200 iterations was performed. The identified metabolites were annotated using the KEGG Compound database (http://www.kegg.jp/kegg/compound/, accessed on 3 December 2023), followed by mapping the annotated metabolites to the KEGG Pathway database (http://www.kegg.jp/kegg/pathway.html, accessed on 3 December 2023). Pathways containing strongly regulated metabolites were subjected to metabolite set enrichment analysis (MSEA), and significance was evaluated using hypergeometric test *p* values.

### 4.6. qRT-PCR and RT-PCR Validation of the Transcriptomic DEGs

We selected eight DEGs associated with ovule development to validate the RNA-Seq results, with the *CmActin* gene utilized as an internal reference [40]. cDNAs were synthesized from the RNA samples used for transcriptome sequencing. The qRT-PCR assays were performed in an AriaMx real-time PCR system (Agilent Technologies, Santa Clara, CA, USA) with the HiScript II One Step qRT-PCR SYBR Green Kit (Vazyme, Nanjing, China), and the detailed procedure was carried out by referring to the instruction manual. Supplementary Table S5 lists all the primers used. The estimation of FC was based on threshold cycles following the 2^−ΔΔCT^ method. Three biological and three technical replicates were assessed. The RT-PCR reference was from Marone et al. [41]. The PCR program was set up as 10 s at 98 °C, 5 s at 60 °C, and 1 min at 72 °C for 20 cycles. The RT-PCR primers used were consistent with those used for qRT-PCR.

### 4.7. Spraying with Exogenous Growth Regulators

To confirm that ethylene, and not the ethylene synthesis precursor (ACC), plays a regulatory role in ovules, we sprayed plants with ethylene, ACC, and the ethylene synthesis inhibitor AIB. Thirty-nine *C. mollissima* ‘*Zunhuaduanci*’ trees in good condition with similar growth and no pests or diseases were selected for spraying with external growth regulators. Four concentrations were tested for ACC, ETH, and AIB (50 mg/L, 100 mg/L, 200 mg/L, and 400 mg/L), and water treatment was used as a control (CK). A one-way, single plant block experimental design was used, with one concentration of a growth regulator sprayed on each tree and replicated three times. Two sprays were applied during the chestnut pollination period (June 6) and the critical period of chestnut fertilization (June 15). A clear and windless evening was chosen for the test, and hanging signs indicated the type and concentration of the sprayed regulator. At maturity (7 September), 10 fruiting mother branches were selected from the east, south, west, and north directions on the treatment plants to determine the single-nut weight, the number of nuts in a single bur, the bur set rate, the empty bur rate, and the longitudinal and transverse diameters of the chestnut fruit.

### 4.8. Data Analysis

The data are expressed as the mean ± standard error (SE) of at least three independent replicates. For metabolite analysis, spraying treatment assays, and qRT-PCR analyses, the data were subjected to one-way analysis of variance (ANOVA) with Duncan’s test in SPSS 19.0 (IBM, Armonk, NY, USA) and Excel 2019 software (Microsoft, SEA, Redmon, WA, USA). The figures were generated with the SigmaPlot 12.5 (Systat Software, Inc., San Jose, CA, USA) software package and Excel 2019 software. The significance of differences between treated and control fruit was evaluated at the *p* < 0.05 level.

## 5. Conclusions

In this study, by analyzing the transcriptomes of ovules in different developmental periods, it was found that DEGs were enriched mainly in carbohydrate metabolism-related pathways, phytohormone signaling, and protein processing in the endoplasmic reticulum pathways during the critical period of fertilization (15 to 18 DAP). The *HSP* genes were enriched mainly in the protein processing in the endoplasmic reticulum pathway, indicating that they were extremely sensitive to temperature during the period of fertilization and that temperature abnormalities led to fertilization failure. Combined transcriptomic and metabolomic data analysis of fertile and abortive ovules from three developmental stages revealed that DEGs and DAMs were enriched in various amino acid metabolism, phytohormone signaling, and energy metabolism pathways in fertile and abortive ovules from three developmental stages, suggesting that hormones and sugars play important roles in ovule development. Therefore, we hypothesized that during the critical period of chestnut ovule fertilization, abnormal temperature leads to disorders associated with the secretion of hormones, especially auxin and ethylene, which in turn leads to abnormal sugar synthesis and catabolism, and ultimately leads to ovule abortion. Therefore, ovule abortion due to abnormal temperatures could be regulated by spraying with ethylene and IAA, thus increasing chestnut yield. By spraying ethylene and ACC in the field, it was found that appropriate concentrations of ethylene and ACC increased the fertility of chestnut ovules. These findings could lead to further research on chestnut ovule abortion and provide breeders with ideas for improving chestnut yield through temperature and hormone regulation.

## Figures and Tables

**Table 1:** Effects of different concentrations of growth regulators on the development of chestnut ovaries.

Type of Growth Regulator	Concentration/(mg ± ^L−1^)	Single-Nut Weight/g	Cross Diameter/mm	Longitudinal Diameter/mm	Side Diameter/mm	Bur Set Rate/%	Empty Bur Rate/%	Number of Nuts Per Bur
ETH	0	10.15 ± 0.860 ab	29.93 ± 0.642 a	29.72 ± 0.981 a	19.13 ± 2.933 bc	81.70 ± 5.586 ab	0	2.22 ± 0.667 b
	50	10.02 ± 1.202 ab	30.32 ± 0.692 a	26.26 ± 1.435 b	21.34 ± 2.406 ab	83.10 ± 4.468 a	0	3.00 ± 0.000 a
	100	10.37 ± 1.465 a	30.10 ± 1.440 a	27.66 ± 1.74 ab	23.34 ± 2.521 a	72.02 ± 5.011 b	0	3.00 ± 0.000 a
	200	9.30 ± 0.624 ab	30.26 ± 1.022 a	28.27 ± 1.02 ab	19.35 ± 2.196 bc	65.30 ± 4.672 c	0	2.60 ± 0.548 ab
	400	8.71 ± 1.323 b	30.86 ± 1.051 a	27.07 ± 2.027 b	17.14 ± 2.581 c	52.69 ± 7.821 c	0	1.25 ± 0.500 c
AIB	0	10.15 ± 0.860 a	29.93 ± 0.642 a	29.72 ± 0.981 a	19.13 ± 2.933 a	81.70 ± 5.586 a	0	2.22 ± 0.667 a
	50	10.22 ± 1.20 a	30.00 ± 1.979 a	27.45 ± 1.553 b	20.85 ± 2.216 a	79.40 ± 5.320 a	0	2.86 ± 0.523 a
	100	10.64 ± 1.325 a	34.31 ± 2.574 a	27.60 ± 0.996 b	18.04 ± 2.955 a	82.40 ± 6542 a	0	2.80 ± 0.447 a
	200	8.87 ± 0.697 a	28.60 ± 1.135 a	26.99 ± 1.421 b	17.25 ± 1.433 a	75.83 ± 9.295 a	0	2.40 ± 0.894 a
	400	9.48 ± 0.754 a	32.26 ± 10.376 a	27.77 ± 0.766 b	19.26 ± 2.384 a	42.40 ± 5.495 b	0	2.43 ± 0.408 a
ACC	0	10.15 ± 0.860 a	29.93 ± 0.642 a	29.72 ± 0.981 a	19.13 ± 2.933 a	81.70 ± 5.586 a	0	2.22 ± 0.667 b
	50	8.16 ± 1.079 b	29.17 ± 1.258 ab	26.72 ± 1.327 b	17.78 ± 1.809 a	84.54 ± 8.303 a	0	3.20 ± 0.447 a
	100	7.54 ± 0.897 bc	28.10 ± 0.698 bc	25.03 ± 1.015 bc	18.37 ± 1.782 a	71.46 ± 11.88 ab	0	2.43 ± 0.535 ab
	200	6.80 ± 0.327 c	27.48 ± 0.273 c	23.38 ± 0.665 bc	17.04 ± 0.212 a	42.75 ± 10.506 bc	0	2.29 ± 0.756 b
	400	6.90 ± 0.551 c	27.13 ± 0.885 c	24.82 ± 1.580 c	16.95 ± 1.498 a	23.32 ± 18.239 c	0	2.00 ± 1.000 b

Note: The data in the table are presented as the mean ± standard deviation (SD). Different lowercase letters in the same column indicate a significant difference at the 0.05 level, and the same letter indicates no significant difference.

## Data Availability

Data are contained within the article and Supplementary Materials.

## Supplementary Materials

The supporting information can be downloaded at: https://www.mdpi.com/article/10.3390/ijms25041974/s1.

## References

[1] Barro-Trastoy D., Dolores Gomez M., Tornero P., Perez-Amador M.A. (2020). On the way to ovules: The hormonal regulation of ovule development. Crit. Rev. Plant Sci..

[2] Chen T., Xie M., Jiang Y., Yuan T. (2022). Abortion occurs during double fertilization and ovule development in *Paeonia Ludlowii*. J. Plant Res..

[3] Niu S., Du X., Wei D., Liu S., Tang Q., Bian D., Zhang Y., Cui Y., Gao Z. (2021). Heat stress after pollination reduces kernel number in maize by insufficient assimilates. Front. Genet..

[4] Backhaus A.E., Griffiths C., Vergara-Cruces A., Simmonds J., Lee R., Morris R.J., Uauy C. (2023). Delayed development of basal spikelets in wheat explains their increased floret abortion and rudimentary nature. J. Exp. Bot..

[5] Zhang D.Y., Gao M., Li S.N. (2021). Advances in the mechanism of plant seed embryo degradation. J. Northeast Agric. Univ..

[6] Dong H., Zhen Z., Peng J., Chang L., Gong Q., Wang N.N. (2011). Loss of ACS7 confers abiotic stress tolerance by modulating ABA sensitivity and accumulation in *Arabidopsis*. J. Exp. Bot..

[7] Xu W.J., Fu Y., Dong H.L., Chen Z.F., Zhang Q.Y., Mao S.X., He Y.J., Qian W. (2014). Morphological identification and physiological characterization of an ovule failure mutant in kale-type oilseed rape. Sci. Agric. Sin..

[8] Zhang S.Y. (2019). Effect of High Temperature Stress on Reproductive Organ Development and Yield of Summer Maize. Master’s Thesis.

[9] Zhao J.R., Chen G.P. (1990). Effects of shading treatment at different stages ofplant development on grain production of corn (*Zea maysl.*) and observations of tip kernal abortion. Sci. Agric. Sin..

[10] Fan X., Yuan D., Tang J., Tian X., Zhang L., Zou F., Tan X. (2015). Sporogenesis and gametogenesis in chinese chinquapin (*Castanea Henryi* (Skam) Rehder & Wilson) and their systematic implications. Trees.

[11] Wang Y.L., Xu Z.H., Li S., Liang Z.M., Xue X.R., Bai T.K., Yang Z.P. (2023). Straw return to field and post-flowering irrigation to improve yield and water and nitrogen use efficiency of spring maize. Sci. Agric. Sin..

[12] Ren H.Y., Gong G.H., Wang Y.K., Zhao A.L., Xue X.F. (2019). Progress of genes related to plant embryo failure. Chin. Agric. Sci. Bull..

[13] Li Z., Jiao Y., Zhang C., Dou M., Weng K., Wang Y., Xu Y. (2021). VvHDZ28 positively regulate salicylic acid biosynthesis during seed abortion in thompson seedless. Plant Biotechnol. J..

[14] Liao S., Wang L., Li J., Ruan Y.-L. (2020). Cell wall invertase is essential for ovule development through sugar signaling rather than provision of carbon nutrients(1[OPEN]). Plant Physiol..

[15] Jung C.J., Hur Y.Y., Jung S.-M., Noh J.H., Do G.R., Park S.J., Nam J.C., Park K.-S., Hwang H.S., Choi D. (2014). Transcriptional changes of gibberellin oxidase genes in grapevines with or without gibberellin application during Inflorescence development. J. Plant Res..

[16] Zhao Z.H., Huang X.L., Li K.C., Liang H.Y., Liang W.H., Liao J.M., Li B.C., Liang X.J., Wen J.H. (2018). Ovule formation and embryonic development of chestnut in guangxi. Guangxi For. Sci..

[17] Du B., Zhang Q., Cao Q., Xing Y., Qin L., Fang K. (2020). Changes of cell wall components during embryogenesis of *Castanea mollissima*. J. Plant Res..

[18] Li L. (2020). Anatomical Observation and Preliminary Study of Molecular Mechanism of Fertilization and Mmbryo Development in Chestnut. Master’s Thesis.

[19] Mou W., Kao Y.-T., Michard E., Simon A.A., Li D., Wudick M.M., Lizzio M.A., Feijó J.A., Chang C. (2020). Ethylene-independent signaling by the ethylene precursor ACC in *Arabidopsis* ovular pollen tube attraction. Nat. Commun..

[20] Yoon J., Cho L.-H., Tun W., Jeon J.-S., An G. (2021). Sucrose signaling in higher plants. Plant Sci..

[21] Ruan Y.L. (2014). Sucrose Metabolism: Gateway to diverse carbon use and sugar signaling. Annu. Rev. Plant Biol..

[22] Ribalta F.M., Pazos-Navarro M., Edwards K., Ross J.J., Croser J.S., Ochatt S.J. (2019). Expression patterns of key hormones related to pea (*Pisum sativum* L.) embryo physiological maturity shift in response to accelerated growth conditions. Front. Plant Sci..

[23] Figueiredo D., Köhler C. (2018). Auxin: A molecular trigger of seed development. Genes Dev..

[24] Liu S., Lei C., Zhu Z., Li M., Chen Z., He W., Liu B., Chen L., Li X., Xie Y. (2023). Genome-wide analysis and identification of 1-Aminocyclopropane-1-Carboxylate Synthase (ACS) gene family in wheat (*Triticum aestivum* L.). Int. J. Mol. Sci..

[25] Woodson W.R., Park K.Y., Drory A., Larsen P.B., Wang H. (1992). Expression of ethylene biosynthetic pathway transcripts in senescing carnation flowers. Plant Physiol..

[26] Zhang X.S., O’Neill S.D. (1993). Ovary and gametophyte development are coordinately regulated by auxin and ethylene following pollination. Plant Cell.

[27] Tsai W.-C., Hsiao Y.Y., Pan Z.-J., Kuoh C.-S., Chen W., Chen H.-H. (2008). The role of ethylene in orchid ovule development. Plant Sci..

[28] Li Z., Howell S.H. (2021). Heat Stress Responses and Thermotolerance in Maize. Int. J. Mol. Sci..

[29] Feng J., Fan P., Jiang P., Lv S., Chen X., Li Y. (2014). Chloroplast-targeted Hsp90 plays essential roles in plastid development and embryogenesis in *Arabidopsis* possibly linking with VIPP1. Physiol. Plant..

[30] Sandhu J., Irvin L., Liu K., Staswick P., Zhang C., Walia H. (2021). Endoplasmic reticulum stress pathway mediates the early heat stress response of developing rice seeds. Plant Cell Environ..

[31] Hatfield J.L., Prueger J.H. (2015). Temperature extremes: Effect on plant growth and development. Weather Clim. Extrem..

[32] Poor P., Nawaz K., Gupta R., Ashfaque F., Khan M.I.R. (2022). Ethylene involvement in the regulation of heat stress tolerance in plants. Plant Cell Rep..

[33] Huang J., Zhao X., Bürger M., Chory J., Wang X. (2023). The role of ethylene in plant temperature stress response. Trends Plant Sci..

[34] Savada R.P., Ozga J.A., Jayasinghege C.P.A., Waduthanthri K.D., Reinecke D.M. (2017). Heat stress differentially modifies ethylene biosynthesis and signaling in pea floral and fruit tissues. Plant Mol. Biol..

[35] Wang S., Li L.-X., Fang Y., Li D., Mao Z., Zhu Z., Chen X.-S., Feng S.-Q. (2022). MdERF1B–MdMYC2 module integrates ethylene and jasmonic acid to regulate the biosynthesis of anthocyanin in apple. Hortic. Res..

[36] Khan M.I.R., Trivellini A., Chhillar H., Chopra P., Ferrante A., Khan N.A., Ismail A.M. (2020). The significance and functions of ethylene in flooding stress tolerance in plants. Environ. Exp. Bot..

[37] Wang Q. (2012). Research on the Impact of Variety Allocation on Fruit Set and Fruit Quality of *Castanea mollissima* ‘Yanshan’. Master’s Thesis.

[38] Liu N., Zhao H., Hou L., Zhang C., Bo W., Pang X., Li Y. (2023). HPLC-MS/MS-based and transcriptome analysis reveal the effects of ABA and MeJA on jujube (*Ziziphus jujuba* Mill.) cracking. Food Chem..

[39] Yuan H., Zeng X., Shi J., Xu Q., Wang Y., Jabu D., Sang Z., Nyima T. (2018). Time-course comparative metabolite profiling under osmotic stress in tolerant and sensitive Tibetan hulless barley. BioMed Res. Int..

[40] Shi L., Wang J., Liu Y., Ma C., Guo S., Lin S., Wang J. (2021). Transcriptome analysis of genes involved in starch biosynthesis in developing chinese chestnut (*Castanea mollissima* Blume) seed kernels. Sci. Rep..

[41] Marone M., Mozzetti S., De Ritis D., Pierelli L., Scambia G. (2001). Semiquantitative RT-PCR Analysis to Assess the Expression Levels of Multiple Transcripts from the Same Sample. Biol. Proced. Online.

