# Supplementary material for: Transcriptomic and Metabolic Profiling Reveal the Mechanism of Ovule Development in Castanea mollissima
Source: Int J Mol Sci. 2024 Feb 6;25(4):1974. doi: 10.3390/ijms25041974 (PMC10888392; doi:10.3390/ijms25041974)
Supplement: Supplementary file 1 [file ijms-25-01974-s001.zip › ijms-2781538-Supplementary Figures.pdf]

Supplementary figure

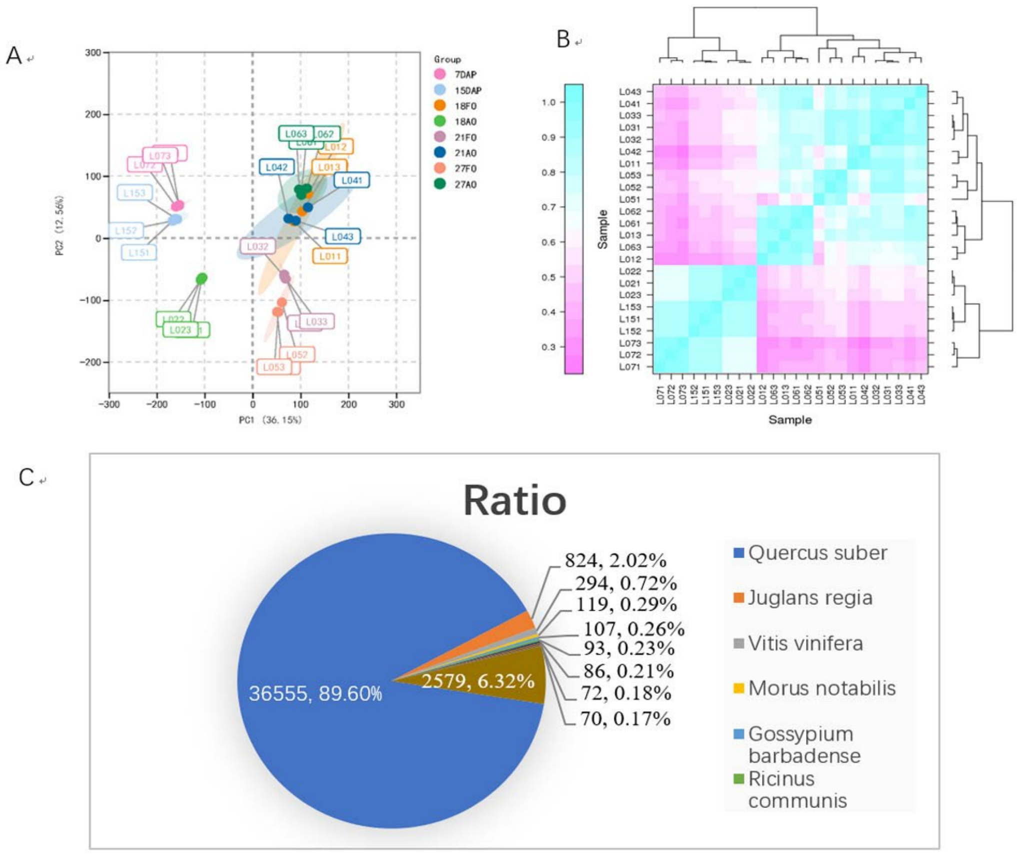

Figure S1. Principal component analysis and correlation analysis of metabolites identified

Note: Clean reads: the total number of pair end reads in clean data; clean bases: total base number of clean data; GC content: the percentage of G and C in clean data; ≥ Q30%: the percentage of bases whose mass value is greater than or equal to 30.

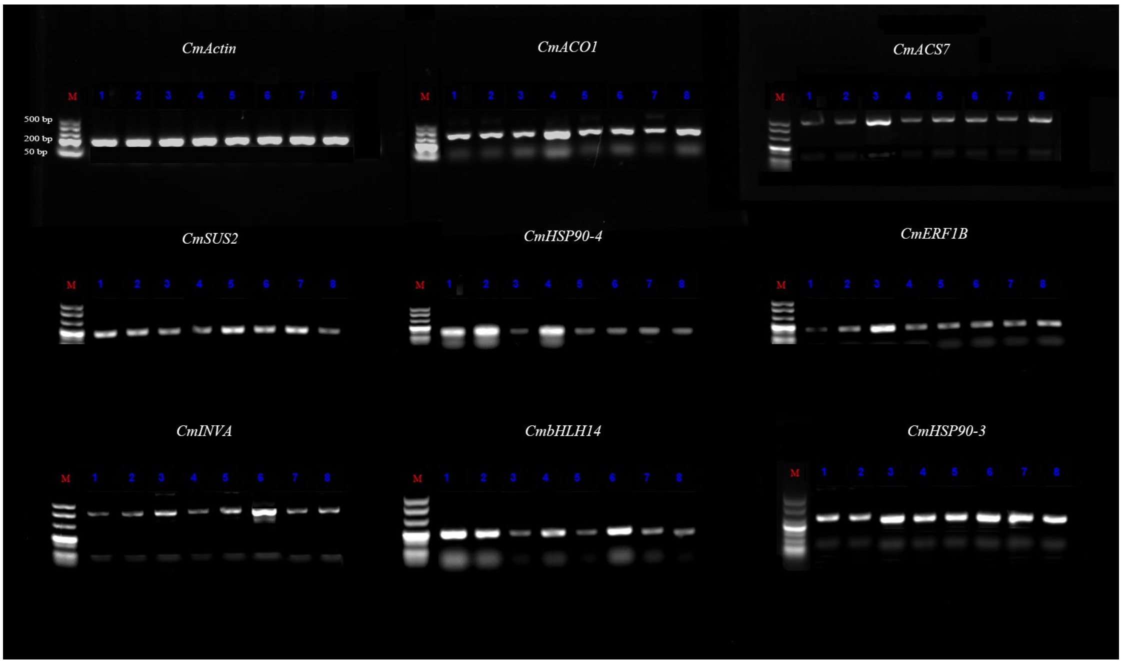

Figure S2 RT-PCR gel electrophoresis

Lane M. 50-500 bp DNA marker; Lane 1. 7 DAP; Lane 2. 15 DAP; Lane 3. 18 FO; Lane 4. 18 AO; Lane 5. 21FO; Lane 6. 21AO; Lane 7. 27FO; Lane 8. 27AO.
